# Homozygous *SALL1* Mutation Causes a Novel Multiple Congenital Anomaly—Mental Retardation Syndrome

**DOI:** 10.1016/j.jpeds.2012.08.042

**Published:** 2013-03

**Authors:** Julia Vodopiutz, Heinz Zoller, Aimée L. Fenwick, Richard Arnhold, Max Schmid, Daniela Prayer, Thomas Müller, Andreas Repa, Arnold Pollak, Christoph Aufricht, Andrew O.M. Wilkie, Andreas R. Janecke

**Affiliations:** 1 Department of Pediatrics and Adolescent Medicine, Medical University Vienna, Austria; 2 Department of Medicine II Gastroenterology and Hepatology, Medical University Innsbruck, Austria; 3 Weatherall Institute of Molecular Medicine, University of Oxford, United Kingdom; 4 Pathologisch-Bakteriologisches Institut, Danube Hospital, Vienna, Austria; 5 Department of Obstetrics and Feto-Maternal Medicine, Medical University Vienna, Austria; 6 Division of Neuroradiology and Musculoskeletal Radiology, Medical University Vienna, Austria; 7 Department of Pediatrics I, Innsbruck Medical University, Innsbruck, Austria; 8 Division of Human Genetics, Innsbruck Medical University, Innsbruck, Austria

**Keywords:** cDNA, Complementary DNA, CNS, central nervous system, CNS-TBS, Central nervous system-Townes-Brocks syndrome, mRNA, messenger RNA, MCA-MR, Multiple congenital anomaly-mental retardation, NMD, Nonsense-mediated mRNA decay, TBS, Townes–Brocks syndrome, TOF, Tetralogy of Fallot

## Abstract

**Objective:**

To delineate a novel autosomal recessive multiple congenital anomaly-mental retardation (MCA-MR) syndrome in 2 female siblings of a consanguineous pedigree and to identify the disease-causing mutation.

**Study design:**

Both siblings were clinically characterized and homozygosity mapping and sequencing of candidate genes were applied. The contribution of nonsense-mediated messenger RNA (mRNA) decay to the expression of mutant mRNA in fibroblasts of a healthy carrier and a control was studied by pyrosequencing.

**Results:**

We identified the first homozygous *SALL1* mutation, c.3160C > T (p.R1054*), in 2 female siblings presenting with multiple congenital anomalies, central nervous system defects, cortical blindness, and absence of psychomotor development (ie, a novel recognizable, autosomal recessive MCA-MR). The mutant *SALL1* transcript partially undergoes nonsense-mediated mRNA decay and is present at 43% of the normal transcript level in the fibroblasts of a healthy carrier.

**Conclusion:**

Previously heterozygous *SALL1* mutations and deletions have been associated with dominantly inherited anal-renal-radial-ear developmental anomalies. We identified an allelic recessive *SALL1*-related MCA-MR. Our findings imply that quantity and quality of *SALL1* transcript are important for SALL1 function and determine phenotype, and mode of inheritance, of allelic *SALL1*-related disorders. This novel MCA-MR emphasizes SALL1 function as critical for normal central nervous system development and warrants a detailed neurologic investigation in all individuals with *SALL1* mutations.

SALL1, a multiple zinc finger transcription factor of 1324 amino acids, is a key mediator of developmental pathways during organogenesis and cell differentiation. *SALL1* regulation and SALL1 protein function are incompletely understood.^1,2^ Heterozygous early-truncating *SALL1* mutations are known to cause autosomal dominant Townes–Brocks syndrome (TBS, MIM #107480), a malformation syndrome without severe central nervous system (CNS) involvement.^3,4^ Such mutations result in truncated proteins thought to act by dominant negative effects.^5,6^ *SALL1* haplo-insufficiency^7^ and late-truncating mutations which partially undergo nonsense-mediated mRNA decay (NMD) cause mild TBS,^5,7,8^ suggesting distinct pathophysiological mechanisms of *SALL1* mutations.

We identified 2 female sibling, born to healthy consanguineous parents, affected by a multiple congenital anomaly-mental retardation (MCA-MR) syndrome with absence of psychomotor development caused by the first identified homozygous *SALL1* mutation c.3160C > T (p.R1054*). The mutation leads to a reduced amount of transcript encoding a late-truncated SALL1. Our results indicate that quantity and quality of residual *SALL1* transcript determine the phenotype and the mode of inheritance of the 2 allelic disorders, TBS and this MCA-MR, and imply SALL1 function as critical for human CNS development.

## Methods

Written informed consent was obtained from all participants, and the study was approved by the ethics committee of Medical University Vienna.

### Patient 1

**Figure 1:**
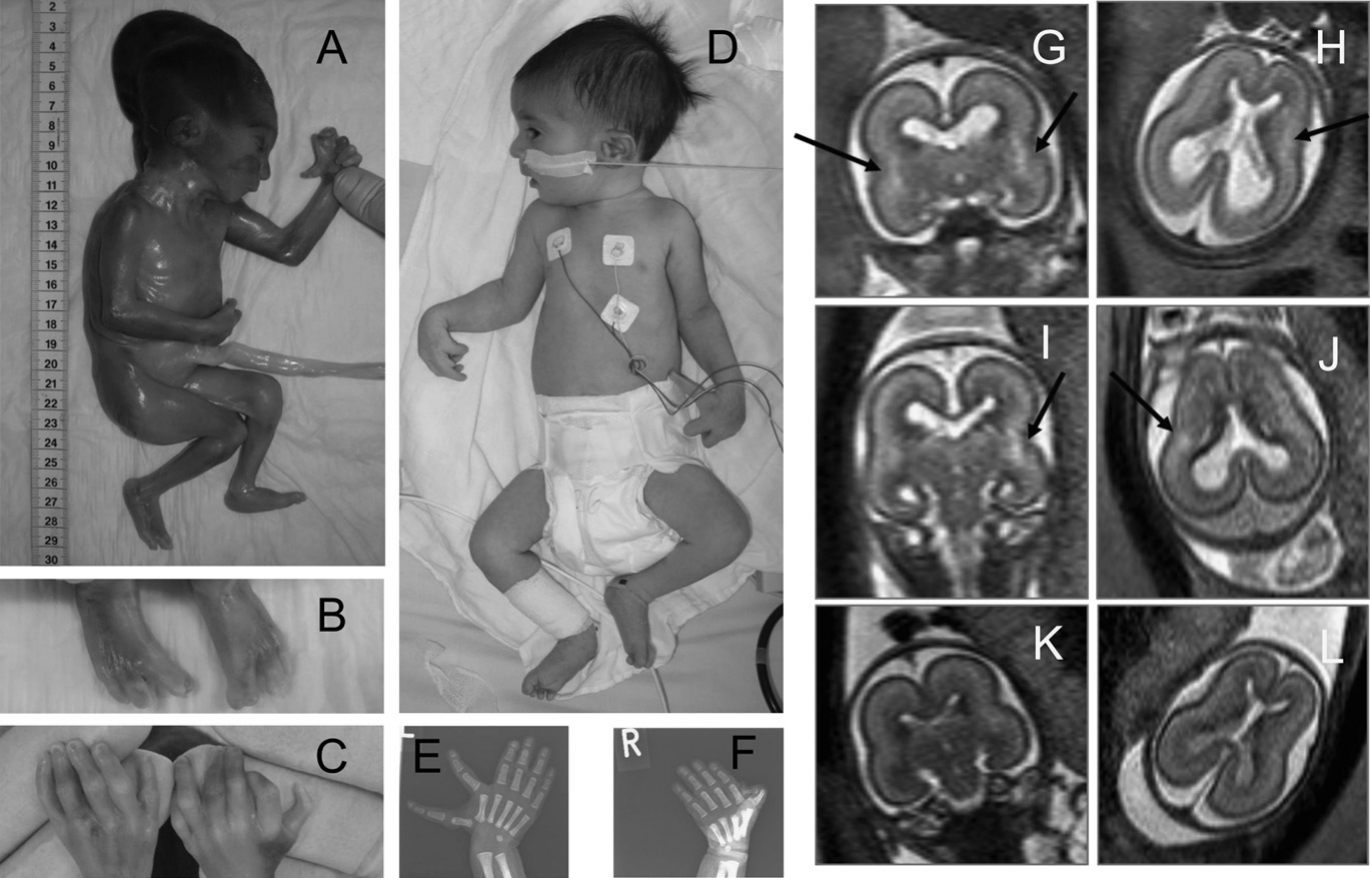
**A-F,** Clinical manifestation and **G-L,** CNS abnormalities in fetal magnetic resonance imaging in **A-C, G, H,** patient 1 and **D-F, I, J,** patient 2. Note **B, E, F,** limb malformations consisting of bilateral triphalangeal thumbs, extra preaxial digit on the left hand, and incomplete preaxial polydactyly on the right hand in both patients and **C,** bilateral cutaneous I/II and III/IV toe syndactyly in patient 1. **G, H,** T2-weighted fast spin-echo sequences in coronal and axial planes of patient 1, **I, J,** patient 2, and **K, L,** a healthy control fetus, each at 21 weeks of pregnancy. Both affected fetuses present with hypoplastic corpus callosum, asymmetric ventricles, and hyperintense T2-weighted signal changes in the region of the triangular crossroads (*arrows* in **G-J**).

In the second pregnancy of a consanguineous Turkish couple, the fetus was diagnosed with corpus callosum hypoplasia and tetralogy of Fallot (TOF) by fetal ultrasound at 21 + 1 weeks, and fetal magnetic resonance imaging revealed oligohydramnios and CNS abnormalities (Figure 1). Diagnosis of a MCA-MR syndrome without further classification was made, and the parents opted for termination of the pregnancy at 22 + 2 weeks. The female fetus's weight was 515 g (10th percentile), crown-heel length 30 cm (50th percentile), and head circumference 19 cm (50th percentile). There were contractures of upper and lower limbs and bilateral ear and limb malformations (Figure 1). Autopsy additionally showed bilateral hypo-plastic multicystic kidneys.

### Patient 2

Patient 2 was the female sibling of patient 1. Fetal ultrasound disclosed a ventricular septal defect at 19 + 5 weeks; hypoplastic kidneys and oligohydramnios later progressing to anhydramnios were noted at 21 weeks. Fetal magnetic resonance imaging at 21 + 1 and 23 + 5 weeks showed hexadactyly of the left hand and CNS abnormalities identical to patient 1 (Figure 1). Abnormal fetal movement patterns were noted. The patient was born at term with Apgar scores 4/6/9, weight 3570 g (50th-70th percentile), length 52 cm (50th-70th percentile), and head circumference 35.5 cm (50th-75th percentile). She presented at birth with TOF, atrial septal defect, imperforate anus with perianal fistula, bilateral hypoplastic multicystic kidneys with chronic renal failure, generalized muscular hypotonia, and ear and hand malformations. Respiratory distress and lung hypoplasia secondary to oligohydramnios required intubation for 48 hours after birth. Chronic renal failure progressed to end-stage kidney disease, requiring dialysis from 7 months of age. Hypotonia and insufficient central coordination required permanent tube feeding. At 6 months of age, TOF was corrected by surgery. Left-sided sensorineural hearing loss and right-sided deafness, respectively, were diagnosed by brainstem audiometry. Ophthalmologic investigations at birth, 3, and 6 months of age revealed absent visual fixation without any structural eye abnormalities, indicating cortical blindness. Fanconi anemia was excluded. At 18 months of age, she showed no head control, was not able to roll over or sit, and showed no social interaction. The patient died at 19 months of age because of bacteremia. Normal karyotypes were obtained in both patients.

### Clinical Investigations in Carriers

Eleven mutation carriers were investigated for dysmorphism, congenital anomalies, and heart sounds, with emphasis on minor TBS features. All were healthy except for a ventricular septal defect in the father of both patients described here, requiring no treatment. In both parents renal ultrasound, skeletal radiographs, and hearing tests were normal. Renal function was normal in both parents and 5 additional disease mutation carriers. One further carrier was not available for clinical investigation but was reported to be healthy. There was no family history of anal atresia, hearing loss, or impaired renal function.

### DNA Analysis, Linkage Analysis, and *SALL1* Sequencing

DNA was extracted from tissue sections of patient 1 and from blood or buccal smear samples of all other participants. For linkage analysis, 2 affected and 10 unaffected relatives were genotyped with Illumina Hap370 single nucleotide polymorphism arrays (Illumina, Little Chesterford, United Kingdom). GeneChip hybridizations and respective processing steps were performed according to the manufacturer's instructions by a professional GeneChip service provider (Microarray Facility Tübingen, Medical Genetics Department, Tübingen, Germany). Multipoint logarithm of the odds scores and haplotypes were obtained with the ALLEGRO program^9^ under the hypothesis of an autosomal-recessive, fully penetrant mutation, inherited identical-by-descent (homozygosity mapping). The *SALL1* coding regions and splice sites were polymerase chain reaction amplified and directly sequenced in patient 2. All family members were tested for the presence of the c.3160C > T mutation detected in patient 2. For *SALL1* sequencing primer sequences were based on the National Center for Biotechnology Information reference entries for messenger RNA (NM_002968) and genomic DNA of *SALL1* (NG_007990.1). Primers and conditions are available from the authors on request.

### RNA Extraction, Cyclohexamide Treatment, and Pyrosequencing

The expression level of mutant relative to wild-type allele, with or without exposure to cycloheximide, was quantified in fibroblast complementary DNA (cDNA) from a heterozygote by pyrosequencing.^5^ A positive control experiment was conducted in parallel on fibroblasts heterozygous for the *SALL1* mutation 3414_3415delAT, which was previously shown to undergo NMD.^5^ Cells were rinsed in sterile phosphate buffered saline (Invitrogen, Paisley, United Kingdom), stored at −70°C in 1 mL of TRIzol (Invitrogen) and RNA extracted according to the manufacturer's protocol. Three micrograms of RNA were used for cDNA synthesis, using random hexamer primers, according to the manufacturer's instructions (RevertAid Premium First Strand cDNA Synthesis Kit; Fermentas, York, United Kingdom).

Pyrosequencing was carried out on a PSQ-HS96A instrument (Biotage, Ystrad Mynach, United Kingdom) to quantify the expression level of mutant allele relative to wild-type allele, with or without exposure to cyclohexamide as described.^5^ Hemi-nested touchdown polymerase chain reaction was used to avoid concurrent amplification of the X chromosomal *SALL1P*, starting with primers spanning intron 2-3 (forward primer 5′-ACACTGCTTGTGACATTTGTGGCAAAAC-3′, reverse primer 5′-TAGAAATGTCATGGGGCCATCCACAGAGAGC-3′) for 16 touchdown cylces (70°C-62°C), followed by 25 cycles at 62°C using the above forward primer and the reverse 5′-B-ACGAAGCCGTTGACCTCTGTCTTGATGA-3′. The pyrosequencing primer was: 5′-ACATGTTGACACATCAGAT-3′, using the dispensation order: G-T-G-A-T-G-A-C-G-A-C-G-A-T-C-G to compare the mutant/wild-type peaks G^3^/G^9^, A^4^/A^10^, G^6^/G^12^, A^7^/A^13^.

The proportion of mutant allele (mutant/wild-type, corrected for background) was first calculated in replicate control samples (genomic DNA from individuals with normal [CC], heterozygous [CT] or homozygous mutant [TT]) genotypes at position 3160 of *SALL1*. The peak comparison G^3^/G^9^ was discarded as it showed a higher standard deviation than the other measurements. Quadratic equations to fit calibration curves for each of the other peak comparisons were individually calculated and the estimated proportions of mutant allele ascertained by obtaining the means of these calculated figures from 3 replicate experiments.

## Results

**Figure 2:**
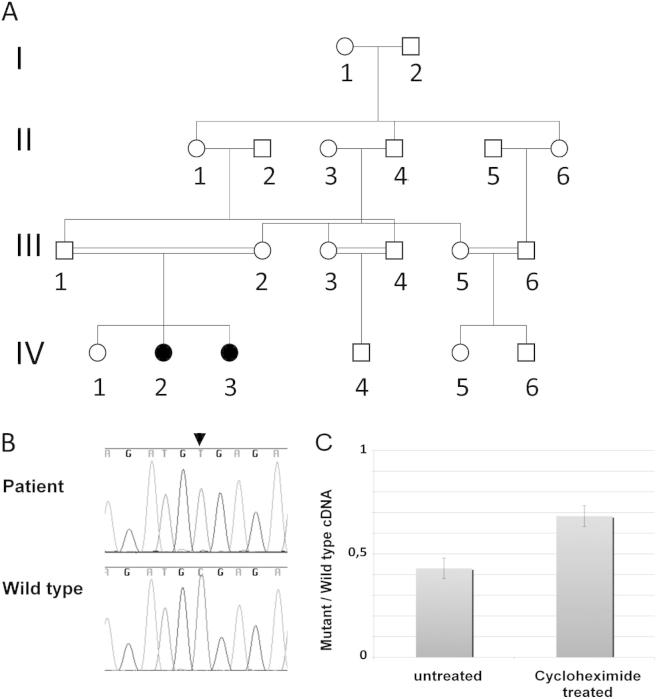
**A,** Simplified pedigree showing multiple consanguinity loops, CNS-TBS patients represented by *filled black circles*. For privacy protection carriers are not indicated. **B,** Sequencing chromatograms from a patient (*top*) and a control (*bottom*). The c.3160C > T mutation is indicated by an *arrowhead*. **C,** Relative quantification by pyrosequencing of the mutant versus the wild-type allele in *SALL1* cDNA from carrier fibroblasts, before and after treatment by cycloheximide.

**Figure 3:**
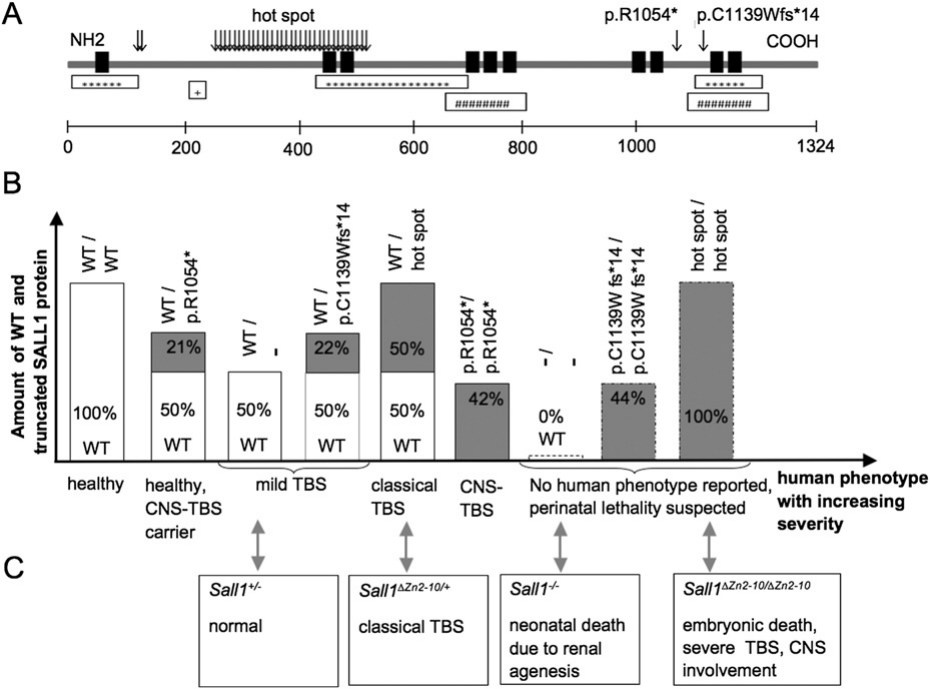
**A,** Schematic of SALL1 protein, localization of known mutations and the best characterized SALL1 interaction domains: SALL1 (1324 aa) harbors 10 zinc finger domains (*black boxes*) and heterochromatin (*), beta-catenin (#), and SALL1,2,3,4 (+) binding sites. In TBS all previously reported mutations are heterozygous truncating mutations (*arrows*). The majority of mutations cluster in a hot spot region and result in 5′-truncation, but late 3′-truncating mutations and heterozygous whole gene deletions are also described in mild TBS. CNS-TBS is caused by a novel homozygous late 3′-truncating mutation p.R1054*. **B,** Amount of wild-type (*white*) and truncated (*gray*) SALL1 protein in relation to phenotypic expression in humans. In the presented model the remaining transcript is assumed to represent the level of protein. Healthy subjects express 50% of the wild-type SALL1 protein per allele. CNS-TBS patients express 21% 3′-truncated SALL1 protein per mutant allele and no wild-type protein due to homozygous p.R1054* mutation which partially undergoes NMD. CNS-TBS carriers are healthy and express 50% wild-type SALL1 protein and 21% 3′-truncated SALL1 protein. All TBS patients express 50% wild-type SALL1 protein from the wild-type allele. Classical TBS patients express additionally 50% 5′-truncated SALL1 protein due to 5′-hot spot mutations which escape NMD. There are 3 patients with mild TBS due to haploinsufficiency caused by heterozygous gene deletion. Patients with mild TBS due to p.C1139Wfs*14 mutation, which partially undergo NMD express additional 22% late-truncated SALL1 protein. **C,** SALL1 mouse models corresponding to **B,** Heterozygous *Sall1*^*ΔZn2-10*^ mice, expressing truncated SALL1 protein, present with a dominant TBS phenotype including limb defects and hearing loss,^14^ whereas *Sall1*^−/−^ mice present with autosomal recessive isolated bilateral renal agenesis and no phenotype in heterozygotes.^13^ The phenotype in both CNS-TBS patients does not correspond to the phenotype in homozygous *Sall1*^−/−^ mice but resembles the more severe phenotype seen in homozygous *Sall1*^*ΔZn2-10*^ mice including renal agenesis (100%), limb defects (57%), anal malformations (>50%), and neural tube closure defects (38%). *WT*, wild-type.

We delineate a novel, autosomal recessive MCA-MR, characterized by TOF, polycystic hypoplastic kidneys with prenatal onset of chronic renal failure, limb and ear malformations, sensorineural deafness, corpus callosum hypoplasia, cortical blindness, and complete lack of psychomotor development (Figure 1), already manifesting prenatally as an abnormal fetal movement pattern. Homozygosity mapping in this consanguineous family (Figure 2, A) revealed a maximal logarithm of the odds score of 1.9 within 5 homozygous intervals located on chromosomes 2, 9, and 16 (data not shown). We selected *SALL1* from the largest candidate region for mutation analysis on the basis of partial clinical overlap with TBS, known function and expression patterns. Sequencing identified a homozygous *SALL1* mutation, c.3160C > T (p.R1054*), in both affected siblings that segregates with the disease (Figure 2, B). Twelve healthy family members were carriers of this mutation. This novel mutation leads to a premature stop codon in exon 2 and was expected to trigger NMD. Pyrosequencing showed that the mutant transcript was present at 43% of the level of the normal transcript in carrier fibroblasts; this increased to 68% after cycloheximide treatment, indicating a partial contribution of NMD to the relative deficiency of mutant transcript (Figure 2, C). The SALL1 protein encoded by the mutant transcript lacks 270 C-terminal residues including the last double zinc finger domain and a beta-catenin binding domain (Figure 3).^1^

## Discussion

The condition we describe here, tentatively termed central nervous system-Townes-Brocks syndrome (CNS-TBS), is distinguished from TBS by prenatal onset of chronic renal failure, severe CNS involvement, and autosomal recessive inheritance. Corpus callosum hypoplasia is rare in TBS, the majority of TBS patients have normal intelligence, and profound mental retardation has not been reported in TBS.^4,10,11^ A search of published reports in Medline, Online Mendelian Inheritance in Men, the London Dysmorphology, and the POSSUM Databases did not reveal any known syndromes resembling the pattern of findings in our patients. We conclude that CNS-TBS represents a distinct clinical entity, which is supported by the demonstration of a previously not described genetic cause in our patients, a novel and homozygous (p.R1054*) *SALL1* mutation.

We show that the mutant transcript partially undergoes NMD, suggesting that a decreased amount of mutant protein is produced. The residual transcript is expected to result in a late-truncated protein. Together, the decreased amount and supposedly limited functionality of SALL1 protein initially imply loss-of-function as the disease mechanism in CNS-TBS. However, complete loss-of-function of 1 *SALL1* allele (ie, haplo-insufficiency resulting from whole gene deletions) already causes mild TBS phenotypes, as emerged from the observation of few patients with such deletions.^7^ Interestingly, no TBS features were present in any of 12 heterozygotes for p.R1054* from the extended pedigree. This observation supports the idea that some protein is produced from the mutant allele. Furthermore, this observation supports the idea that the truncated protein resulting from p.R1054*, thus, must preserve some degree of SALL1 function, apparently sufficient to prevent heterozygotes from TBS features. However, the residual SALL1 function resulting from the p.R1054* mutation is not sufficient to prevent homozygotes for this mutation from having disease; homozygotes for this mutation are supposed to produce less functional SALL1 protein than individuals with only 1 copy of *SALL1*, as they are much more severely affected. In addition, the truncated p.R1054* protein does not seem to exert overt dominant-negative effects in p.R1054* heterozygotes. In contrast, a mild TBS phenotype is observed in patients with reported coding mutations just 3′ of the p.R1054* mutation in *SALL1*. Three of these mutations represent frameshifting-truncating mutations that remove the same 2 protein domains as the p.R1054* mutation does.^10^ A comparable reduction in transcript level was found for 1 of these mutations (c.3414_3415delAT, p.C1139Wfs*14).^5,8,10^ We hypothesize that mild TBS symptoms in these patients likely arise by qualitative, dominant-negative effects of such truncated proteins. We hypothesize that the distinctly abnormal C-termini of residual SALL1 proteins determine any dominant-negative effects, comparable with the example of distinct phenotypic outcome associated with receptor tyrosine kinase *ROR2* mutations.^12^ The presence or absence of such effects might be determined by degree of NMD, protein stability, and nuclear and cytoplasmic distribution of different truncated mutant isoforms of SALL1 (Figure 3). In summary, we conclude that heterozygotes for the p.R1054* *SALL1* mutation are unaffected as the truncated p.R1054* protein preserves some degree of residual SALL1 function and does not exert overt dominant-negative effects, both sufficient to prevent p.R1054* heterozygotes from TBS features. We consider the ventricular septal defect in 1 carrier for CNS-TBS to be unrelated to his being heterozygous for the p.R1054* *SALL1* mutation, as it is represents an isolated, mild defect, and such heart defects are very common in the general population; however, we cannot exclude that this ventricular septal defect represents a mild phenotype with incomplete penetrance of this late-truncating *SALL1* mutation.

The balance between quantity and quality of wild-type and truncated SALL1 protein suggests a model in which the phenotypic outcome of *SALL1*-related disorders is determined by an integration of loss-of-function and dominant negative effects. This model accounts for classical TBS, in which early-truncated and wild-type SALL1 proteins are expressed at equal levels.^5^ Through homodimerization of mutant and wild-type SALL1, a reduction of functional protein at natural target sites might cause disease by loss-of-function, and additional dominant-negative effects are mediated by heterodimerization with other members of the SALL family.^6^ The hypothesis of an integration of loss-of-function and dominant negative effects is supported by *Sall1* mouse models. Homozygous *Sall1-*deficient mice present with renal agenesis at birth,^13^ and heterozygotes are normal. Heterozygous *Sall1*^*ΔZn2-10*^ mice, expressing early truncated Sall1, resemble the classical TBS phenotype and homozygous *Sall1*^*ΔZn2-10*^ mice are embryonically lethal and present with a severe TBS phenotype with CNS involvement.^6,14^ The phenotype in p.R1054* homozygotes resembles the phenotype of homozygous *Sall1*^*ΔZn2-10*^ mice. We, therefore, hypothesize that in p.R1054* homozygotes the truncated protein exerts additional dominant-negative or gain-of-function effects. The profound neurologic phenotype in humans and mice with homozygous expression of truncated SALL1 protein implicates an important function of SALL1 in neurogenesis.^15^ Functional redundancy of Sall1 and Sall4 in murine neurulation may explain the apparent lack of a CNS phenotype in Salll1-/- mice^16^ and heterozygous *Sall1*^*ΔZn2-10*^ mice. Considering multiorgan involvement, absence of any psychomotor development and death in early infancy in p.R1054* homozygotes, CNS-TBS might constitute the most severe viable *SALL1*-related phenotype in humans (Figure 3).

Most of the *SALL1* mutations reported to-date escape NMD either completely or partially,^5,6^ which remains unexplained. Escaping NMD^17^ can modify phenotypes conveyed by allelic truncating mutations, as demonstrated for mutations implicated in neurologic disorders in *SOX10*,^18^ *MPZ*,^19^ and *NFIX*.^20^ We observed an unusually low restoration of mutant messenger RNA expression upon protein synthesis inhibition. While this finding remains currently unexplained, it emphasizes the fact that the classical NMD mechanism does not apply to most or all *SALL1* mutations.

Our results imply that both quantity and quality of residual transcript might be important for residual SALL1 function and determine the phenotype and the mode of inheritance of the 2 allelic disorders TBS and CNS-TBS. *SOX10*,^18^ *ROR2*,^12^ and *NFIX*^20^ are examples of other genes in which mutations cause different recessive or dominant conditions, with partially overlapping phenotypes. CNS involvement in CNS-TBS highlights an essential role of SALL1 in human neurogenesis and warrants a detailed neurologic investigation in all patients with *SALL1* mutations.
